# Supplementary material for: Predicting visual function by interpreting a neuronal wiring diagram
Source: Nature. 2024 Oct 2;634(8032):113–23. doi: 10.1038/s41586-024-07953-5 (PMC11446822; doi:10.1038/s41586-024-07953-5)
Supplement: Supplementary file 6 — CRF and ERF predictions for individual TmY4 and TmY9 cells. Analogous to Supplementary Data 3, but for TmY target types. Shown are the top four monosynaptic pathways, the strongest pathway passing through each of the top ten intermediary types (ranking from Extended Data Fig. 7), and the trisynaptic pathway Tm1–TmY–Dm3–TmY (see the section entitled Prediction of spatial normalization). [file 41586_2024_7953_MOESM6_ESM.zip › DataS4/TmY9q/720575940613626777.pdf]

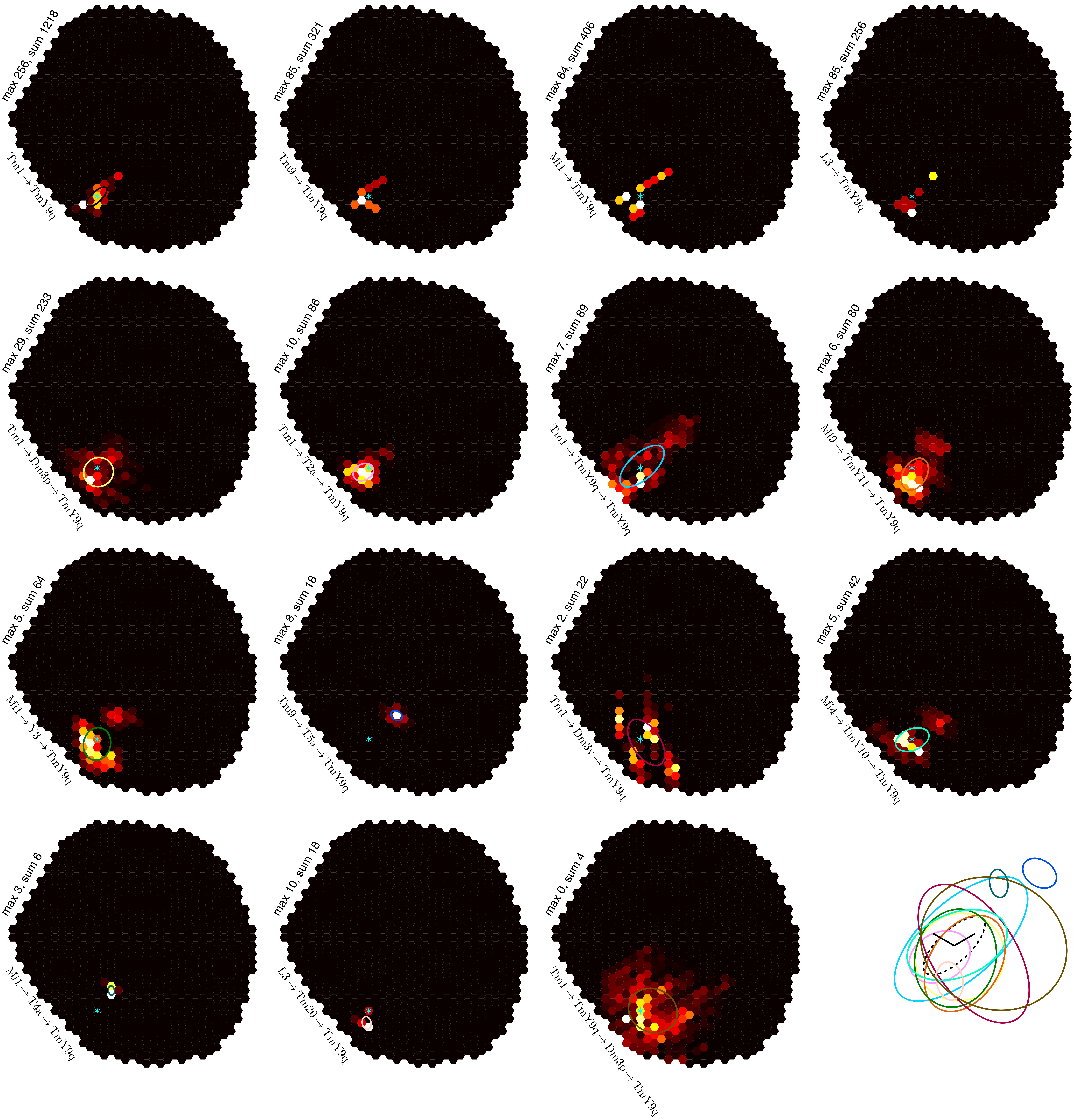

max 256, sum 1218
Tm1 → TmY9q
max 85, sum 321
Tm9 → TmY9q
max 64, sum 406
Mi1 → TmY9q
max 85, sum 256
L3 → TmY9q
max 29, sum 233
Tm1 → Dm3p → TmY9q
max 10, sum 86
Tm1 → T2a → TmY9q
max 7, sum 89
Tm1 → TmY9q → TmY9q
max 6, sum 80
Mi9 → TmY11 → TmY9q
max 5, sum 64
Mi1 → Y3 → TmY9q
max 8, sum 18
Tm9 → T5a → TmY9q
max 2, sum 22
Tm1 → Dm3v → TmY9q
max 5, sum 42
Mi4 → TmY10 → TmY9q
max 3, sum 6
Mi1 → T4a → TmY9q
max 10, sum 18
L3 → Tm20 → TmY9q
max 0, sum 4
Tm1 → TmY9q → Dm3p → TmY9q
